# The risk factors of postoperative nausea and vomiting in patients undergoing laparoscopic sleeve gastrectomy and laparoscopic distal gastrectomy: a propensity score matching analysis

**DOI:** 10.1038/s41598-023-34992-1

**Published:** 2023-05-15

**Authors:** Peng Chen, Rongrong Du, Zhengyao Chang, Wenxing Gao, Wen Zhao, Lujia Jin, Yingjie Zhao, Dingchang Li, Hao Liu, Xianqiang Liu, Guanglong Dong

**Affiliations:** 1 Medical School of Chinese PLA, Beijing, China; 2 Department of General Surgery and Institute of General Surgery, The First Medical Center of Chinese, PLA General Hospital, Beijing, 100853 China; 3 School of Medical, Nankai University, Tianjin, 300071 China

**Keywords:** Gastric cancer, Cancer, Biomarkers, Diseases

## Abstract

Postoperative nausea and vomiting (PONV) is a common side effect after laparoscopic surgery. The aim of the study is to investigate the variables that could predict PONV in patients who underwent laparoscopic gastrectomy. We divided patients who underwent laparoscopic gastrectomy into PONV and No-PONV groups. Propensity score matching (PSM) was applied to adjust confounding factors for further validation, and ordinal logistic regression analysis was used to identify predictors for PONV. In the ordinal logistic regression analysis, the preoperative neutrophil-to-lymphocyte ratio (NLR) (odds ratio [OR]: 3.19, 95% confidence interval [CI]: 1.38–7.38; *p* < 0.01) was identified as an independent risk factor for the presence of PONV and a predictor of the severity of PONV (OR: 3.44, 95% CI: 1.67–5.20; *p* < 0.01) in 94 PSM patients. Besides, NLR was positively correlated with the PONV score (r = 0.534, *p* < 0.001). In the receiver-operating characteristic (ROC) curve analysis, an NLR with an optimal cutoff value of 1.59 predicted severe PONV with a sensitivity of 72% and specificity of 81%. The NLR was an independent risk factor for the presence of PONV, and a high NLR tends to be positively associated with the severity of PONV after laparoscopic gastrectomy.

Postoperative nausea and vomiting (PONV) is a common side effect after laparoscopic surgery. Guidelines for the management of PONV mention a range of risk factors for PONV, including female, and young adults, risky surgery, use of volatile anesthetics, history of PONV or motion sickness, non-smoking status, postoperative opioids and prolonged duration of anesthesia^1^.In the literature, the morbidity and risk elements for PONV vary by surgery type, anesthesia characteristics, anesthesia duration, postoperative opioid use, anesthetics, and inflammation.

PONV after laparoscopic sleeve gastrectomy (LSG) may be explained by higher gastric intraluminal pressure (ILP) that results from a decrease in the distensibility and compliance of the postoperative gastric pouch compared to that of the whole stomach preoperatively. While PONV after laparoscopic distal gastrectomy (LDG) mainly resulted from different types of gastrointestinal reconstruction compared to the whole stomach preoperatively^2^. Although studies enabled anesthetists to assess PONV risk in individual patients and then develop appropriate anesthetics regimens^3^, it is hard to reduce PONV due to developing appropriate surgery regimens for surgeons.

As a significant contributor to the initiation and aggravation of central obesity, inflammation, regulated by cytokines, could accelerate autocrine and endocrine effects on metabolic health, ultimately promoting whole-body insulin sensitivity and central obesity^4^. In addition to this, increased values of leukocytes and subtypes, such as monocytes, neutrophils, and lymphocytes related to tumor progression^5^. Further studies suggested that the neutrophil to lymphocyte ratio (NLR) was related to patients with gastric cancer and tuberculosis^6,7^. The literature suggested that NLRP3 inflammasome affected the secretion of interleukin-1β (IL-1β) in adipose tissue macrophages (ATMs)^8^. Lee et al. have suggested that PONV after subtotal gastrectomy with Billroth I reconstruction of patients with gastric cancer was only 8%^2^. However, PONV is one of the most common postoperative complications of LSG, patients without antiemetic prophylaxis could incur morbidity rates as high as 80%^3^. Clinical studies have suggested that inflammatory variables collected preoperatively and postoperatively also effectively predicted PONV in pneumothorax patients^9,10^. Moreover, there is no objective measure for the evaluation of PONV after laparoscopic gastrectomy. Therefore, we designed this study to investigate whether the preoperative NLR is an independent risk factor for PONV and contributes to patient risk stratification. To the best of our knowledge, this is the first report on the relationship between NLR and PONV in laparoscopic gastrectomy.

## Materials and methods

### Study population

A comprehensive review of three hundred ninety-two participants who experienced LSG or laparoscopic distal gastrectomy (LDG) at the general surgery department of Chinese PLA General Hospital was retrospectively analyzed between January 2012 and June 2020. This included 100% of the primary LSG procedure within our single center. The study was approved by the ethical review committee of Chinese PLA General Hospital (S2021-247-02). As the study was retrospective, all patients signed a written informed consent for data analysis. It is not necessary to obtain formal consent due to the retrospective nature of the study. Our study inclusion criteria included: (1) body mass index (BMI) > 35 kg/m^2^, or BMI 27.5–34.9 kg/m^2^ with type 2 diabetes with poor glycemic control after lifestyle changes and medication, or with two or more other metabolic diseases; (2) gastric cancer was based on pathological diagnosis of the primary lesion, and with Billroth I reconstruction; (3) the patient with gastric cancer had performed gastric resection with negative margins and modified D2 lymphadenectomy; (4) vital organs such as the heart and lungs that could tolerate laparoscopic surgery; and (5) complete clinical and follow-up data. Exclusion criteria were as follows: (1) patients with severe preoperative hepatic or renal insufficiency, cardiopulmonary insufficiency, or other serious illnesses who could not receive standard treatment; and (2) patients with a previous history of psychiatric or gastrointestinal surgery.

After surgery, patients are transported to the recovery room for assessment of vital signs (temperature, heart rate, blood pressure, and oxygen saturation) and then transported to the general ward. In the ward, patients had a PONV score for nausea and vomiting. The PONV score was determined according to WHO criteria. Class I: no nausea or vomiting; Class II: mild nausea, abdominal discomfort, but no vomiting; Class III: significant vomiting, but no vomiting of stomach contents; Class IV: severe vomiting, with vomiting of stomach juices and other contents that are difficult to control without medication. Postoperative subjects who experienced nausea or vomiting received antiemetic medicines, including intravenous ondansetron 4 mg, or metoclopramide 10 mg, granisetron 0.1 mg, or tropisetron 0.5 mg, according to the physician's decision taking into account the severity of PONV. Patients with postoperative pain were given tramadol 10 mg intramuscular (i.m.), parecoxib 40 mg (i.v.), flurbiprofen 50 mg (i.v.), and morphine 5 mg or 10 mg (i.m.), depending on the severity of the pain. Experienced and certified bariatric surgeons from the same surgical team performed all operations.

Patients are encouraged to walk around and drink water the day after surgery to speed up recovery rather than having a nasogastric tube routinely placed. The study was conducted in accordance with the Declaration of Helsinki (as revised in 2013). Data was collected via a secure web based form housed at the Chinese PLA General Hospital and was provided to the primary investigators compliant database.

### Inflammatory measures

Blood samples were collected within 72 h before surgery and 24 h after surgery. The NLR was calculated by dividing the absolute count of neutrophils by that of lymphocytes.

### Anesthesia

Anesthesia was managed following a standardized clinical protocol. Anesthesia was induced with isoproterenol, remifentanil, sufentanil, succinylcholine, or atracurium, and the patient was then intubated. Anesthesia was maintained with infusions of propofol, remifentanil, and oxygen. Dexamethasone and ondansetron were routinely administered during the final stage of the operation according to PONV prophylaxis guidelines^1^. Isoflurane and volatile anesthetic gases were not used in the process.

### Outcome measurements

Nausea is an upset in the stomach that can lead to vomiting. It is usually a prodromal vomiting symptom but may also occur alone, mainly characterized by apparent discomfort in the upper abdomen. Retching was defined as the attempt to bring up stomach contents through the mouth without actually doing so. Vomiting refers to the spillage of food or sputum from the stomach and out of the mouth^11^. The risk factors for postoperative nausea are almost identical to those for vomiting. Therefore, this study did not consider events of nausea or vomiting as separate outcomes. Both vomiting and retching were considered emetic events. This study focuses on the first 48 h after surgery.

### Statistical analysis

Continuous variables were defined as the mean ± standard deviation or median (interquartile range); categorical variables were expressed as percentages. For continuous variables, the Kolmogorov‒Smirnov test was applied to test the normality of distribution, t test, or the Mann‒Whitney U test; a one-way ANOVA model was used to compare. For categorical variables, the chi-square test was used. The Spearman rank test was used to test correlations. A Receiver operating characteristic (ROC) curve analysis was performed to verify the diagnostic accuracy of the NLR level in the presence and severity of PONV. Multivariate and ordinal logistic regression analysis was used to assess the independent predictors and severity of PONV, respectively. A propensity score matching (PSM) was done using a multivariable logistic regression model based on: age, sex, body mass index, smoking, hospital stay, type 2 diabetes mellitus (T2DM), gastroesophageal reflux disease (GERD), PONV history, motion history, neutrophil-to-lymphocyte (NLR), monocyte-to-lymphocyte (MLR), laparoscopic sleeve gastrectomy (LSG), laparoscopic distal gastrectomy (LDG), patient-controlled intravenous analgesia (PCIA), opioid, amidoamine, and ondansetron. Pairs of 392 patients were derived using 1:1 greedy nearest neighbor matching within a PS score of 0.2. After propensity score matching (PSM)^12^, the balance of measured variables between groups was analyzed using a paired t test for continuous measures and the McNemar test for categorical variables. Statistical analyses were performed using SPSS 26.0. Statistical significance was defined as a 2-tailed *p* < 0.05.

### Ethics approval and consent to participate

The authors are accountable for all aspects of the work in ensuring that questions related to the accuracy or integrity of any part of the work are appropriately investigated and resolved. The study was conducted in accordance with the Declaration of Helsinki (as revised in 2013). The study was approved by the ethical review committee of Chinese PLA General Hospital (S2021-247-02).

## Results

### Relationship between NLR and PONV before and after PSM

**Figure 1:**
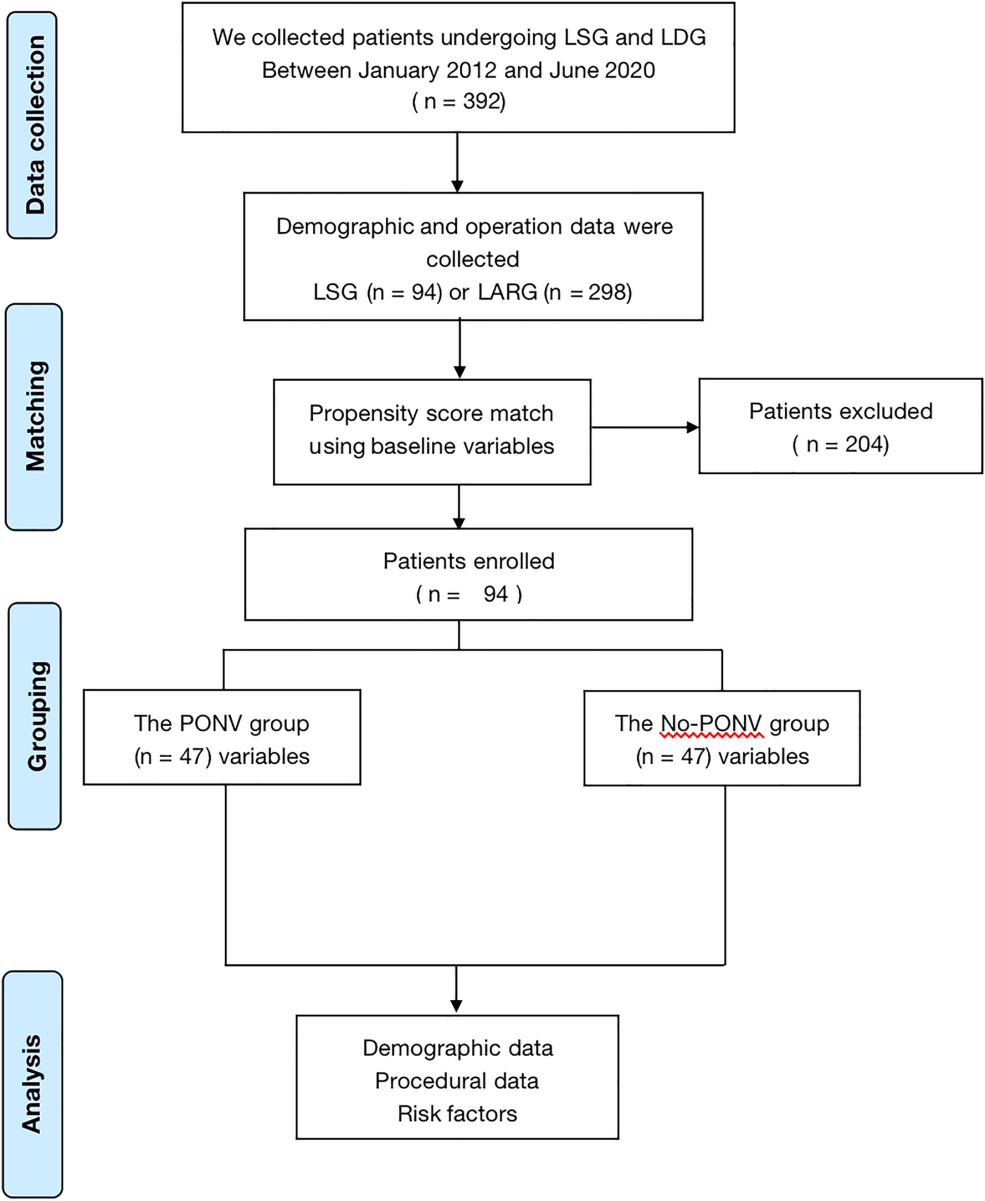
Flow diagram of this study.

A total of 392 patients were enrolled in the study before the PSM process, and 94 matched subjects were selected from each group after 1:1 PSM, effectively counterpoising the preoperative confounding factors for both groups. The study flowchart is shown in Fig. 1. general conditions and perioperative factors for all patients before and after PSM are shown in Table 1. According to WHO criteria for PONV, we divided the patients into the PONV group (n = 108) and the No-PONV group (n = 284). There was a significant statistical difference between PONV with age (*p* < 0.01), sex (*p* < 0.01), BMI (*p* < 0.01), gastroesophageal reflux disease (GERD) (*p* < 0.01), motion history (*p* < 0.01), NLR (*p* < 0.01), monocyte-to-lymphocyte ratio (MLR) (*p* < 0.01), operation (*p* < 0.01), operation time (*p* < 0.01), opioids (*p* < 0.01), and ondansetron (*p* < 0.01) before PSM. There was a significant difference in age and NLR between the PONV group and the no-PONV group after PSM. Lastly, 94 individuals were chosen for further analysis.

**Table 1:** Demographical characteristics and clinical data of the patients before and after PSM.

Variables	Before PSM			After PSM		
	PONV group (n = 108)	No-PONV group (n = 284)	*p* Value	PONV group (n = 47)	No-PONV group (n = 47)	*p* Value
Demographics						
Age	33 (25–45)	60 (41–73)	** < 0.01**	38 (27–66)	62 (41–74)	** < 0.01**
Gender (male%)	44 (41%)	193 (68%)	** < 0.01**	26 (55%)	22 (47%)	0.42
BMI (kg/m^2^)	34 (25–44)	24 (22–27)	** < 0.01**	27 (23–41)	24 (22–27)	0.20
Smoking (%)	64 (60%)	146 (51%)	0.16	29 (62%)	25 (53%)	0.41
Hospital stay	10 (9–13)	11 (9–13)	0.43	11 (9–13)	11 (9–13)	0.26
T2DM (%)	41 (38%)	120 (42%)	0.44	19 (40%)	24 (51%)	0.31
GERD (%)	47 (44%)	60 (21%)	** < 0.01**	17 (36%)	23 (49%)	0.22
PONV HIS(%)	92 (85%)	223 (79%)	0.12	39 (83%)	41 (87%)	0.57
Motion HIS(%)	82 (76%)	177 (62%)	** < 0.01**	35 (74%)	30 (64%)	0.27
Laboratory test						
NLR	2.0 (1.6–2.9)	1.7 (1.3–2.3)	** < 0.01**	1.8 (1.5–2.2)	1.7 (1.3–2.3)	** < 0.01**
MLR	1.9 (1.5–2.2)	1.6 (1.3–2.2)	** < 0.01**	1.8 (1.3–2.0)	1.6 (1.3–2.2)	0.81
Operation			** < 0.01**			0.22
LSG	70 (65%)	24 (8%)		18 (38%)	24 (51%)	
LDG	30 (35%)	260 (92%)		29 (62%)	23 (49%)	
Operation time	167 (142–220)	202 (165–240)	** < 0.01**	175 (150–210)	202 (165–243)	0.63
PCIA	91 (84%)	221 (78%)	0.14	36 (77%)	32 (68%)	0.36
Opioid	70 (65%)	55 (19%)	** < 0.01**	21 (45%)	16 (34%)	0.29
Anisodamine	44 (41%)	100 (35%)	0.31	20 (43%)	15 (32%)	0.30
Ondansetron	57 (53%)	13 (5%)	** < 0.01**	13 (28%)	13 (28%)	1.00

*PONV* postoperative nausea vomiting; *PSM* propensity score matching; *BMI* body mass index; *T2DM* type 2 diabetes mellitus; *GERD* gastroesophageal reflux disease; *PONV HIS* PONV history; *Motion HIS*, motion history; *MLR* monocyte-to-lymphocyte; *NLR* neutrophil-to-lymphocyte; *LSG* laparoscopic sleeve gastrectomy; *LDG* laparoscopic distal gastrectomy; *PCIA* patient-controlled intravenous analgesia.

Significant values are given in Bold.

**Figure 2:**
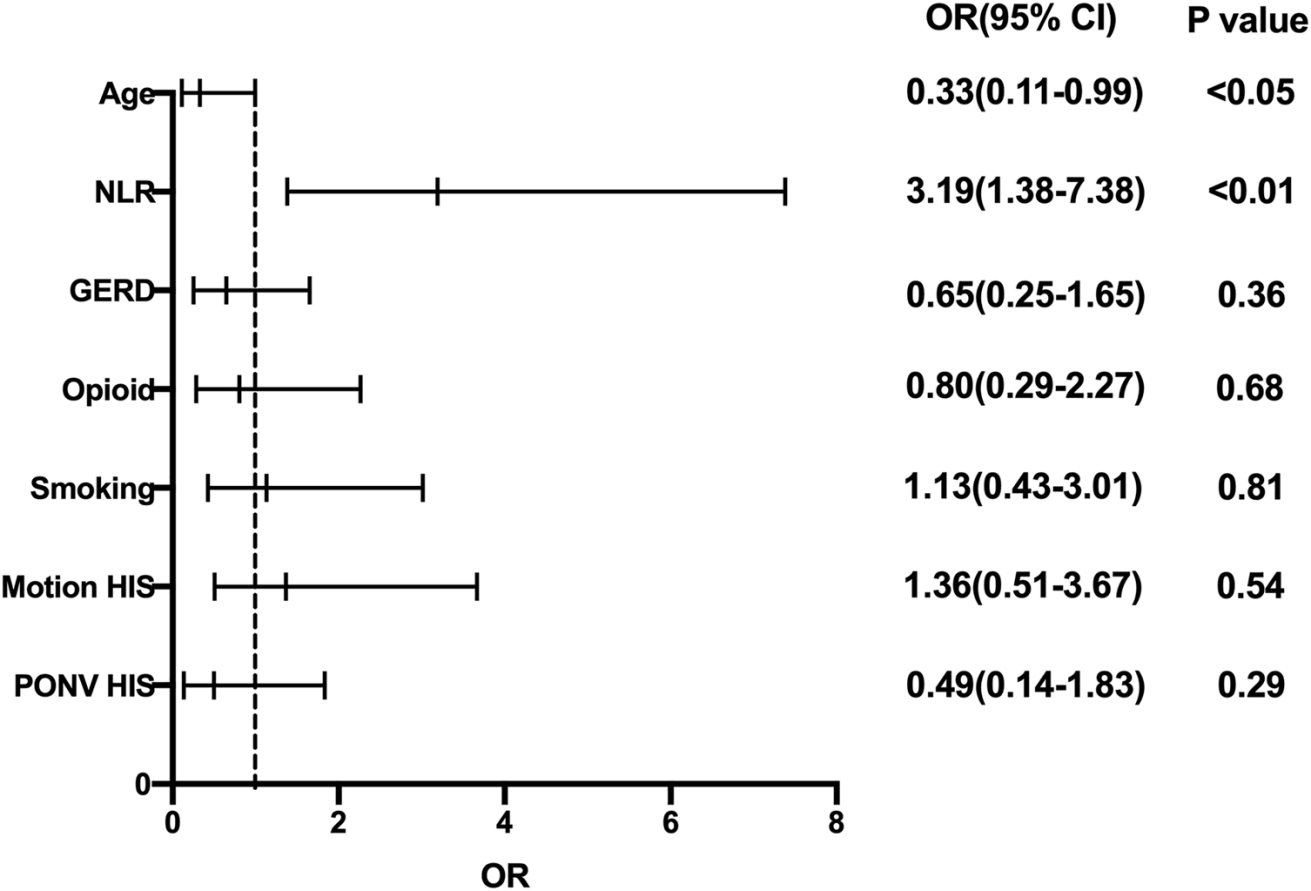
Multivariate logistic regression analysis to assess predictors of PONV after PSM. CI, confidential interval; OR, odds ratio.

Multivariate logistic regression analysis was used to assess 11 clinicopathological characteristics: age, sex, BMI, GERD, motion history, NLR, MLR, type of operation, operation time, opioids, and ondansetron. The result showed that NLR (OR: 3.19, 95% CI: 1.38–7.38,* p* < 0.05) was a risk factor for PONV in patients after PSM; age (OR: 0.33, 95% CI: 0.11–0.99,* p* < 0.05) was a protective factor for PONV in patients after PSM, as shown in Fig. 2.

### The NLR efficacy for detecting PONV after PSM

**Figure 3:**
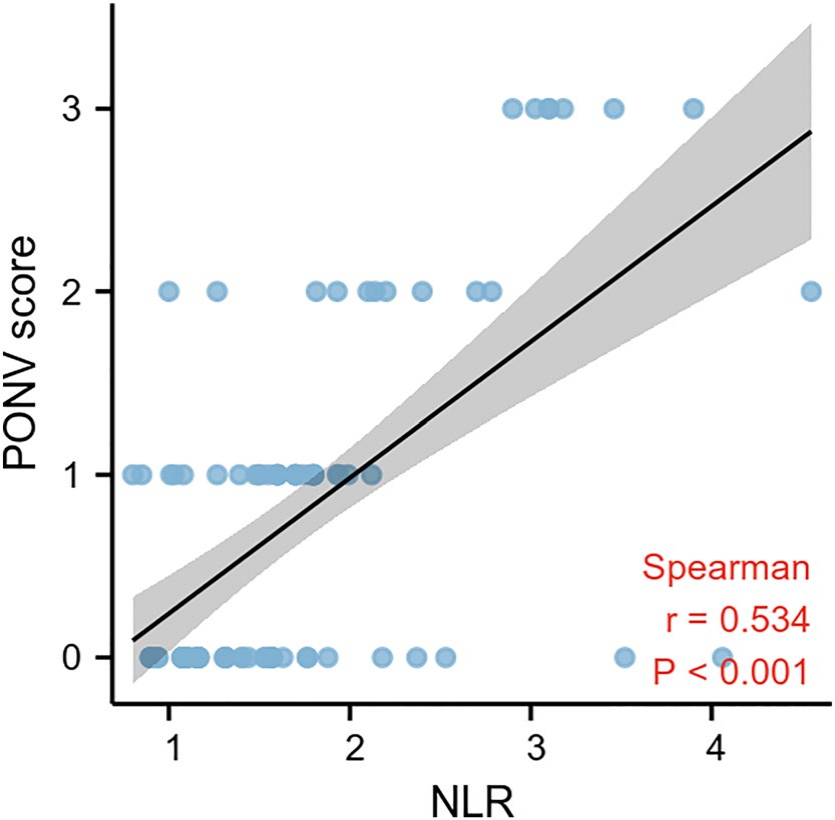
The correlation between the NLR and PONV score.

**Figure 4:**
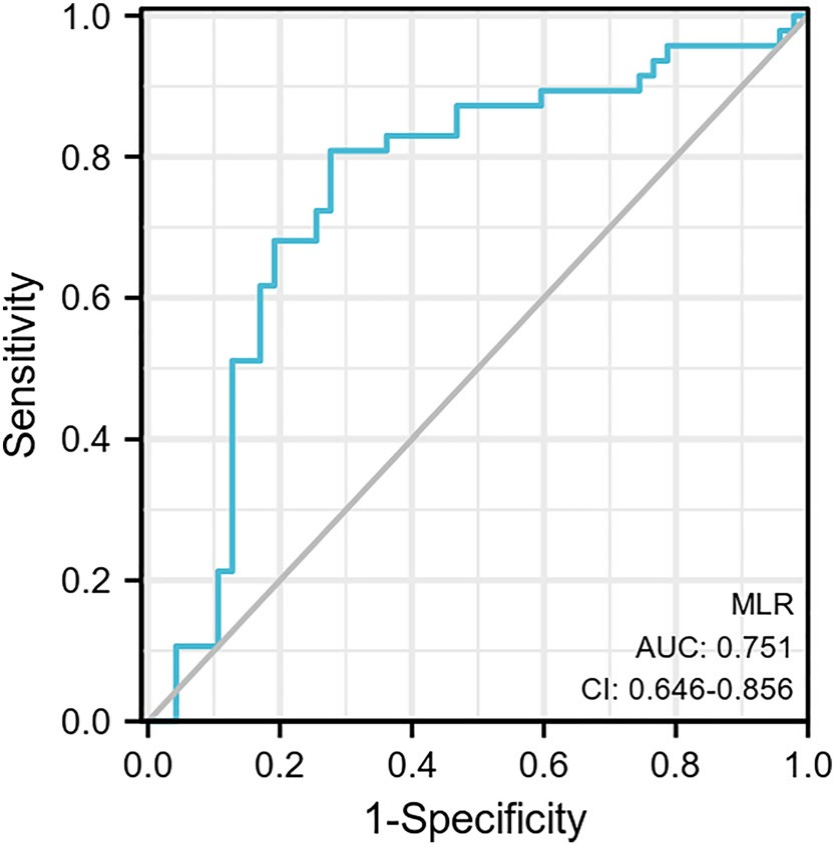
Diagnostic accuracy of the NLR in patients with PONV was analyzed by ROC curves.

A ROC curve analysis was applied to test the efficacy of NLR in predicting PONV with an AUC of 0.751 (95% CI: 0.646–0.856) (Figs. 3, 4). With a cutoff level of 1.59, the NLR predicted PONV with a sensitivity of 72% and specificity of 81%.

### Characteristics of the study population at the baseline according to the severity of PONV after PSM

Based on the PONV score used to assess the severity of PONV, 94 PONV patients were divided into three groups (PONV score: mild = 1, moderate = 2, severe = 3). The control group comprised 47 patients without PONV after PSM, as shown in Table 1. The patient clinicopathological feature distribution is shown in Table 2.

**Table 2:** Baseline characteristics of the study population based on PONV score after PSM.

Variables	Control (n = 47)	Mild (n = 29)	Moderate (n = 11)	Severe (n = 7)	*p* Value
Demographics					
Age	35 (26–44)	38 (22.5–66.5)	31 (25–35)	45 (33–76)	** < 0.05**
Gender(%male)	22 (47%)	16 (55%)	4 (36%)	6 (86%)	0.18
BMI (kg/m^2^)	36.8 (25.0–42.4)	26.6 (24.6–41.0)	33.3 (23.1–47.1)	25.4 (19.7–26.9)	0.17
Smoking (%)	25 (53%)	12 (48%)	6 (55%)	5 (71%)	0.75
Hospital stay	10 (8–11)	9 (9–13)	10 (10–14)	10 (9–12)	0.16
T2D (%)	24 (51%)	13 (45%)	4 (36%)	2 (29%)	0.62
GERD (%)	23 (49%)	16 (64%)	6 (55%)	1 (14%)	0.21
PONV HIS (%)	41 (87%)	25 (86%)	7 (64%)	7 (100%)	0.14
Motion HIS (%)	30 (64%)	21 (72%)	10 (91%)	4 (57%)	0.29
Laboratory test					
NLR	1.16 (1.08–1.57)	1.60 (1.44–1.80)	2.14 (1.81–2.70)	3.10 (3.03–3.46)	** < 0.01**
MLR	1.67 (1.38–2.11)	1.47 (1.16–1.87)	1.94 (1.85–2.02)	2.11 (1.65–2.87)	0.31
Operation (LSG%)	24 (51%)	10 (34%)	7 (64%)	1 (14%)	0.11
Operation time	165 (150–210)	180 (162–215)	157 (120–190)	180 (150–270)	0.21
PCIA	32 (68%)	19 (66%)	10 (91%)	7 (100%)	0.13
Opioid	16 (34%)	9 (31%)	7 (64%)	5 (71%)	0.07
Anisodamine	15 (32%)	10 (34%)	6 (55%)	4 (57%)	0.35
Ondansetron	13 (28%)	3 (10%)	8 (73%)	2 (29%)	** < 0.01**

Significant values are given in Bold.

**Figure 5:**
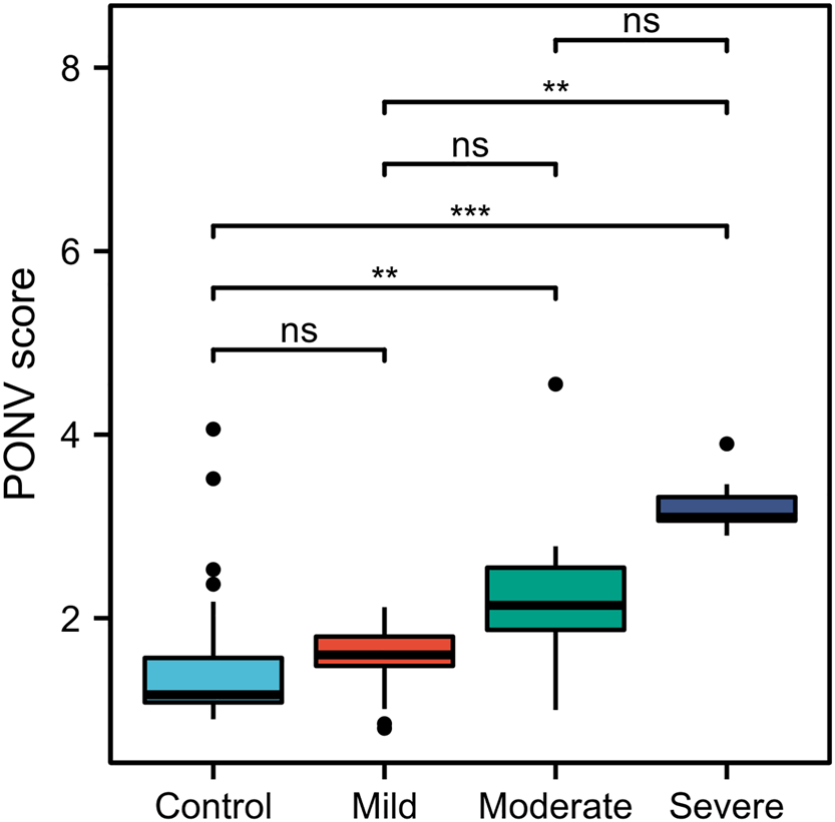
NLR values in propensity score matched 94 colorectal cancer patients according to the PONV score.

There was a significant statistical difference between PONV with age (*p* < 0.05), NLR (*p* < 0.01), and ondansetron (*p* < 0.01). The NLR level in the severe PONV group was statistically higher than in the control and mild group (*p* < 0.01, Fig. 5).

### The NLR is an independent risk factor for the severity of PONV

**Figure 6:**
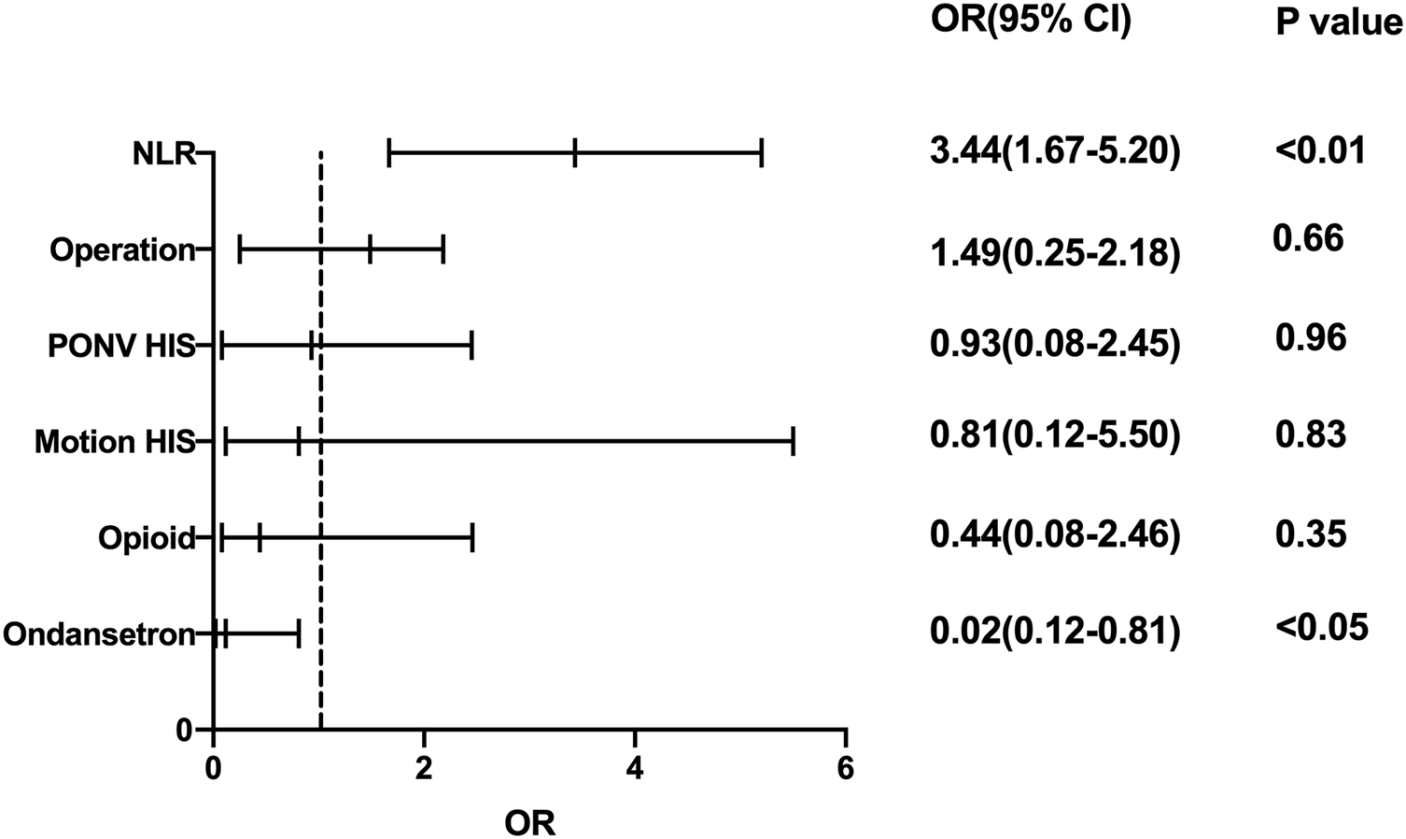
Ordinal logistic regression analysis to assess predictors of PONV after PSM.

Ordinal logistic regression was carried out to investigate which factors could be beneficial for predicting the severity of PONV. The regression analysis results in Fig. 6 demonstrated that NLR (OR: 3.44, 95% CI: 1.67–5.20,* p* < 0.01) was an independent risk factor for the severity of PONV. Ondansetron was an independent protective predictor of the PONV severity (OR: 0.02, 95% CI: 0.12–0.81,* p* < 0.01). In the correlation analysis, the NLR significantly associated with the PONV score (r = 0.534, *p* < 0.01, Fig. 3).

## Discussion

Our study showed that the NLR was significantly higher in patients with PONV and that the NLR was an independent risk factor for PONV after the laparoscopic gastrectomy. In addition, an elevated NLR tends to be positively associated with the severity of PONV after the laparoscopic gastrectomy. Clinical observations suggest excessive corticosteroid levels are related to severe metabolic disorders leading to abdominal obesity^13^. Although corticosteroids are critical for maintaining lipid homeostasis, excess corticosteroids result in reduced eosinophils, increased neutrophils, and decreased lymphocytes. Therefore, this could be explained the pathogenesis between the preoperative NLR and PONV. A clinical observation demonstrated that the higher NLR group had a higher proportion of PONV, so that the NLR could be a marker for PONV, and antiemetic prophylaxis can be given by evaluating the NLR^14^. Another observation showed that the NLR could be used to assess the severity of PONV^15^. Therefore, we assume that NLR might be a predictor for PONV.

It has been almost universally known that adipose tissue is responsive to nutrient overload through an immune response. The original trigger for inflammation is still unclear. Additionally, the injury site has been shown to release various signaling molecules that lead to the recruitment of neutrophils. As the inflammatory response progresses, neutrophils and lymphocytes accumulate at the site of inflammation to neutralize harmful substances^5^. Therefore, this could be explained the pathogenesis between the postoperative NLR and PONV.

It is commonly accepted that LSG is the best treatment for obesity and related comorbidities^16^. Thus, in our study, it was challenging to reduce the preoperative NLR before LSG. Still, we could prevent the morbidity of PONV by using some drugs according to the level of the preoperative NLR. PONV prevention drugs can be used for treatment: dexamethasone, ondansetron, droperidol, aprepitant, diphenhydramine, scopolamine, and dexmedetomidine. In addition, the effect of combined medication on the prevention of PONV was significantly better than that of a single drug.

The present study found that approximately 28% of the patients had PONV after LSG or LDG, among which 65% developed PONV after LSG, while only 30% developed PONV after LDG. However, the incidence of PONV showed no statistical differences between LSG and LDG after PSM. But age is a protective factor against PONV, consistent with Apfel et al.^17^.

The high incidence of PONV in our team might be attributed to reinforcement with a running suture in the margin of the stomach. In contrast, the running suture creates more temporal constriction of the gastric lumen, subsequently increasing nausea and vomiting. Moreover, with the tolerant contraction and diastole of the pyloric sphincter and pyloric ring, the gastric ILP is higher^18^. However, LDG preserves the fundus and resects the pylorus, which may explain the superior distensibility and compliance of the gastric pouch after LDG compared to LSG. Charles et al. suggested that vagal nerves are one of the leading peripheral afferent pathways for triggering PONV^19^. LSG and LDG both involve surgery of the stomach and incisions through branches of the vagus nerve, even if the larger branches along the lesser curvature are less damaged. This direct surgical trauma could contribute to the higher rate of PONV that patients experienced in this study. Additionally, hormones and transmitters may play a significant role as catalysts for PONV in morbidly obese patients during bariatric surgery^20^. However, there was no statistical difference between LSG and LDG after PSM. More samples and more multicenter randomized controlled trials are also required to determine the pathophysiology mechanisms between the two types of surgery.

Ondansetron is the most frequently applied and investigated 5-HT3 receptor antagonist and is believed to be the "gold standard" of PONV therapy^21^. It inhibits the vagus nerve in the central nervous system and intestinal mucosa to reduce nausea and vomiting^22^. Moreover, Ondansetron has similar effectiveness to dexamethasone 4–8 mg^23^. Current evidence supports using multimodal antiemetics to treat post-discharge nausea and vomiting (PDNV). The use of intravenous ondansetron alone after hospital discharge was compared with intravenous dexamethasone, intravenous ondansetron, and ondansetron tablets in an RCT study and reported a significantly lower incidence of PDNV in the latter^24^. Other studies have also compared ondansetron monotherapy with ondansetron plus NK1 receptor antagonists and reported that combination therapy was correlated with a markedly lower incidence of PDNV^25,26^.

In comparison, haloperidol plus dexamethasone was associated with a lower incidence of PDNV compared with both drugs alone^27^. Evidence also suggests that gene polymorphisms and gene expression variants may influence antiemetic effects^28^. Our study confirmed that ondansetron was a protective factor of PONV in ordinal logistic regression. Still, based on the PONV score, the use of ondansetron in the severe group of PONV was much lower in the moderate group, which mainly resulted in the patients combined using other antiemetics prophylaxis, such as metoclopramide.

There are some limitations in the study. First of all, the study is respective and single-center. Secondly, the PONV symptoms were based on the patient’s self-reported symptoms. The proportion with symptoms may be misestimated due to the patients understanding. Additionally, further efforts on nausea are subjective and difficult to measure and standardize across all patients. Therefore, a standardized, validated nausea scale could serve as an objective metric for quantifying PONV in patients.

## Conclusion

NLR was an independent risk factor for the presence of PONV, and high NLR tends to be positively associated with the severity of PONV after surgery.

## Data Availability

All data generated or analyzed during this study are included in this published article. The data that support the findings of this study are available from the corresponding author upon reasonable request.
